# Optimal Analgesic Volume for Popliteal Plexus Block After Total Knee Arthroplasty: A Blinded RCT Protocol

**DOI:** 10.1111/aas.70057

**Published:** 2025-05-10

**Authors:** Johan Kløvgaard Sørensen, Mikkel Schjødt Heide Jensen, Ulrik Grevstad, Lone Nikolajsen, Charlotte Runge

**Affiliations:** ^1^ Department of Anaesthesiology Elective Surgery Centre, Silkeborg Regional Hospital Silkeborg Denmark; ^2^ Department of Clinical Medicine Aarhus University Aarhus Denmark; ^3^ Department of Anaesthesia Gentofte Hospital Gentofte Denmark; ^4^ Department of Anaesthesiology Aarhus University Hospital Aarhus Denmark

**Keywords:** peripheral nerve block, postoperative pain, regional anesthesia, total knee arthroplasty

## Abstract

**Background:**

Adjunct treatment with a popliteal plexus block (PPB) provides moderate enhancement to multimodal analgesic regimens following total knee arthroplasty while maintaining motor function. However, the optimal anesthetic volume of local anesthetic for PPB remains unknown.

**Aim:**

To compare the analgesic effects of PPB with 10 versus 20 mL of local anesthetics as an adjunct to a femoral triangle block after total knee arthroplasty.

**Methods:**

This blinded, controlled, randomized clinical trial will include 120 patients, randomly assigned to receive PPB with either 10 or 20 mL of bupivacaine 5 mg/mL. All patients undergo primary total knee arthroplasty under spinal anesthesia and receive a multimodal analgesia regimen, including paracetamol, ibuprofen, opioids, dexamethasone, and a femoral triangle block.

**Outcomes:**

Primary outcome is 24‐h postoperative opioid consumption. Secondary outcomes include the frequency of patients with opioid‐free analgesia in the first 24 h after surgery, postoperative pain intensity at rest and during mobilization, postoperative muscle function of the leg, ability to mobilize with crutches 6 h after surgery, and Quality of Recovery‐15 survey at 24 h after surgery.

**Conclusion:**

This trial hopes to optimize postoperative pain management after total knee arthroplasty by providing valuable insights into the optimal analgesic volume for PPB.

**Trial Registration:** Danish Data Protection Agency: 693807, clinicaltrials.gov: NCT06908837, CTIS: 2024‐520204‐26‐00

**Abbreviations**
mL: milliliters
MME: morphine milligram equivalents
PPB: popliteal plexus block
REDCap: Research Electronic Data Capture software
TKA: total knee arthroplasty

## 1 Introduction

Primary total knee arthroplasty (TKA) is often associated with severe postoperative pain, highlighting the importance of multimodal analgesia in pain management. Mul helps reduce pain and opioid use, prevent complications, and enhance patient satisfaction [1, 2]. Peripheral nerve blocks are recommended for this multimodal analgesia [3, 4].

The popliteal plexus block (PPB) is a newer peripheral nerve block technique used to enhance multimodal analgesia following TKA. The PPB targets sensory genicular nerves in the popliteal fossa which innervate the posterior aspects and intra‐articular structures of the knee. The PPB complements peripheral nerve blocks for the antero‐medial aspects of the knee, such as the adductor canal block or femoral triangle block. Clinical trials have demonstrated that adjunct treatment with PPB can reduce pain scores and postoperative opioid consumption after TKA without impairing the motor function of the leg [5, 6, 7, 8]. Sørensen et al. [5, 8] reported a 24‐h opioid reduction after TKA equivalent to 15–25 mg of oral morphine with the use of 10 mL of local anesthetic for PPB, whereas Jiang et al. observed a reduction equivalent to 45 mg with 20 mL. Notably, neither study reported significant motor impairment. This motor‐sparing efficacy of the PPB was recently confirmed in a study evaluating the motor effects of 10, 20, and 30 mL of local anesthetic in healthy volunteers [9]. Collectively, these findings suggest a favorable analgesic profile for PPB using larger volumes of local anesthetic without compromising motor function. As the optimal analgesic volume for PPB remains unknown, further randomized clinical trials are necessary.

This trial aims to assess the analgesic effect of PPB with 10 versus 20 mL of local anesthetic as an adjunct to femoral triangle block in a multimodal analgesic regimen following primary unilateral TKA. We hypothesize that PPB with 20 mL of local anesthetic reduces 24‐h postoperative opioid consumption compared with 10 mL after TKA.

## 2 Methods

### 2.1 Trial Design, Randomization, Monitoring, and Approvals

**FIGURE 1:**
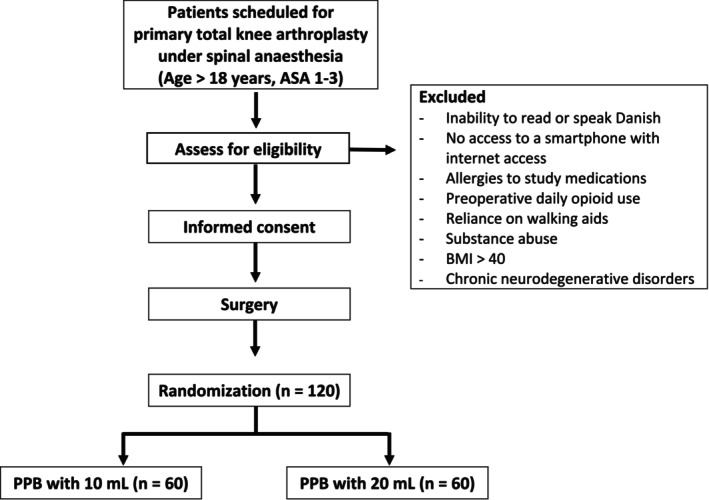
Flow diagram of enrolment. Abbreviations: ASA, American Society of Anaesthesiologists physical status classification; BMI, body mass index; *n*, number of patients; PPB, popliteal plexus block.

This single‐center, blinded, controlled, randomized clinical trial will be conducted at Silkeborg Regional Hospital, Denmark. The trial follows a parallel design (Figure 1), with one group receiving a PPB with 10 mL of bupivacaine 5 mg/mL and the other group receiving a PPB with 20 mL of bupivacaine 5 mg/mL, in addition to multimodal analgesia following TKA. A total of 120 patients will be included and allocated in a 1:1 ratio using computer‐generated block randomization (blocks of 10), managed by an independent data manager in REDCap. The trial will be monitored by the Good Clinical Practice Unit at Aarhus University Hospitals, conducted according to the Declaration of Helsinki and presented in adherence to the Consolidated Standards of Reporting Trials statement [10]. The trial is approved by the Medical Research Ethics Committees and the Danish Medicines Agency (CTIS‐number: 2024‐520204‐26‐00) and registered at the Danish Data Protection Agency and at clinicaltrials.gov (NCT06908837).

### 2.2 Inclusion and Exclusion Criteria

Patients eligible for inclusion are adults over 18 years scheduled for primary TKA under spinal anesthesia, able to provide written informed consent, and classified as American Society of Anaesthesiologists physical status 1, 2, or 3. Exclusion criteria are inability to read or speak Danish, lack of access to a smartphone with internet, allergies to study medications, preoperative consistent daily opioid intake, reliance on walking aids, substance abuse, BMI over 40, or a diagnosis of neurodegenerative disorders.

### 2.3 Blinding

Anesthesiologists performing the peripheral nerve blocks are not blinded to treatment allocation; consequently, they are excluded from data collection and analysis.

All patients, outcome assessors, data analysts, and other personnel involved in the treatment of the patient will be blinded. Patients are shielded from observing their legs and the ultrasound monitor during the nerve block procedure by an opaque cloth hanging at chest level. Furthermore, patients are unlikely to sense the nerve block procedure, as it is performed postoperatively, whereas the effect of spinal anesthesia persists. Even after the spinal anesthesia wears off, patients are likely unable to distinguish between the injection of 10 and 20 mL of bupivacaine for the PPB. The independent data manager blinds group status, dates, and patient‐identifiable data before sending the dataset to the blinded principal investigator, who will perform the statistical analysis. Conclusions on all outcome analyses will be completed with blinding intact before unblinding the dataset.

### 2.4 Outcomes

The primary outcome is total opioid consumption from *T*_0_ (end‐of‐surgery time) to *T*_24_ (24 h after *T*_0_), expressed in oral morphine milligram equivalents (MME).

Secondary outcomes include:

- The frequency of patients with opioid‐free analgesia from *T*_0_ to *T*_24_.
- Pain at rest at *T*_6_ (6 ± 1 h after *T*_0_) and at *T*_24_.
- Pain during a 10‐m walk with crutches at *T*_6_ and *T*_24_.
- The frequency of patients unable to walk 10 m with crutches at *T*_6_.
- The frequency of manual muscle test (MMT) < Grade 3 for dorsiflexion and plantarflexion of the ankle and knee extension at *T*_6_.
- Patient‐reported quality of recovery (Quality of Recovery‐15) at *T*_24_.

### 2.5 Surgical Procedure and Multimodal Analgesia Plan

Patients will undergo primary unilateral TKA with insertion of a non‐cruciate retaining standard cemented prosthesis by a parapatellar medial approach, without the use of a tourniquet or local infiltration analgesia [11].

All patients will receive tablets of paracetamol 1 g and ibuprofen 400 mg 1 h before surgery. Spinal anesthesia is performed with 3 mL of ropivacaine 5 mg/mL. Intravenous dexamethasone 12 mg will be administered perioperatively. Standard treatment with fluids and mild sedation using propofol will be provided at the discretion of the anesthetist. Postoperatively, tablets of paracetamol 1 g are administered four times a day and ibuprofen 400 mg three times a day. After discharge from the post‐anesthesia care unit, the patient will be provided with morphine tablets for self‐administration. The tablets allow the patients to take 5 mg morphine at a time, with a maximum oral dose of 100 mg within the first 24 h after end‐of‐surgery time. If needed, hospital staff can administer rescue doses of intravenous morphine, which will be recorded in the patient file.

### 2.6 Peripheral Nerve Block Procedure

Four 10‐mL syringes are prepared with bupivacaine 5 mg/mL from the department's standard stock and brought to the nerve block procedure. Upon the patient's arrival in the post‐anesthesia care unit after surgery, the anesthesiologist will perform a femoral triangle block using 15 mL of bupivacaine. Subsequently, digital randomization will be conducted in REDCap, followed by administration of the PPB with either 10 or 20 mL of bupivacaine. Any remaining bupivacaine is discarded along with the needle to ensure that the administered volume remains blinded to the patient and clinical staff.

A 22‐gauge, 80‐mm block needle (Temena, Felsberg, Germany) is used for the procedure, guided by a Sonosite X‐Porte ultrasound system (Sonosite; Bothell, WA, USA). For the PPB, a 5–2 MHz curved array transducer (C 60xp; Sonosite) is used, whereas a high‐frequency 15–6 MHz linear array transducer (HFL 50; Sonosite) is used for the femoral triangle block.

#### 2.6.1 The PPB Technique

The superficial femoral artery is identified in the adductor canal and traced caudally until it deviates from the sartorius muscle and is positioned adjacent to the posteromedial margin of the vastus medialis muscle, close to the adductor hiatus. The anatomical landmark for this position is typically 2‐5 cm cranially to the base of patellae. The needle will be inserted medially to the transducer and advanced with an in‐plane technique to the endpoint of injection located on top of the superficial femoral artery, posteromedial to the fascia of the vastus medialis muscle. We will inject the allocated volume of bupivacaine, ensuring anterolaterally spread to the artery [5].

#### 2.6.2 The Femoral Triangle Block Technique

From the apex of the femoral triangle, we trace the superficial femoral artery cranially until the saphenous nerve and nerve to vastus medialis are visualized lateral to the femoral artery, and the intermediate femoral cutaneous nerve (IFCN) is visualized superficial to the sartorius muscle. This optimal ultrasonographical image is typically found 5–10 cm distal to the inguinal crease. The needle will be inserted lateral to the transducer and advanced in‐plane, piercing the sartorius muscle to the endpoint of injection lateral to the femoral artery. Here, we will inject 10 mL of bupivacaine, allowing for repositioning of the needle tip to ensure a spread around both the saphenous nerve and nerve to vastus medialis. Then, the needle tip will be retracted to the superficial side of the sartorius muscle and repositioned to inject 5 mL of bupivacaine into the “flat fat‐filled tunnel” surrounding the IFCN [12].

### 2.7 Sample Size

The sample size was calculated using an online calculator (Georgiev G.Z., “Sample Size Calculator,” https://www.gigacalculator.com/calculators/power‐sample‐size‐calculator.php).

The calculation was based on the mean 24‐h opioid consumption of 33.6‐mg oral MME with a standard deviation of 31.8, as found in our previous trial using a PPB with 10 mL after TKA [5]. A superiority design with a one‐sided hypothesis test, no superiority margin, *α* = 0.05, 1−*β* = 0.8, and a minimal detectable effect of 15‐mg oral MME indicated that 56 patients per group were required. Sixty patients per group will be included to account for dropouts and potential variability.

### 2.8 Study Period, Data Collection, and Analyses

The study period begins on the day of surgery, after signing the informed consent form prior to the surgery. The study period ends 24 h after the end‐of‐surgery time (=*T*_24_). The length of the patient's hospitalization during the study period is determined by their agreement with the surgeon. Patients are allowed to be discharged during the study period.

Pain intensity will be assessed using the 11‐point Numeric Rating Scale (NRS) ranging from 0 = *no pain* to 10 = *worst imaginable pain*. Preoperative baseline pain intensity at rest and during a 10‐m walk will be obtained on the day of surgery.

Prior to assessment of postoperative walking ability with crutches, muscle function is evaluated using the MMT for dorsiflexion and plantarflexion of the ankle, as well as knee extension. MMT results will be graded from 5 to 0 [13]:

5 = active motion against gravity with maximum resistance from assessor.

4 = active motion against gravity with moderate resistance from assessor.

3 = active motion against gravity without resistance.

2 = active motion with gravity eliminated or minimal assistance from assessor.

1 = no active motion, but palpable or observable muscle contraction.

0 = no active motion or muscle contraction.

At *T*_24_, patients will be sent an automated text message including a survey link. In the survey, patients will report their intake of the dispensed morphine tablets, pain intensity at rest and during a 10‐m walk with crutches, and complete the Quality of Recovery‐15 questionnaire [14]. Total opioid consumption will be reported as oral MME, combining hospital‐administered opioid doses from the patient file and self‐reported intake of dispensed morphine tablets.

Total opioid consumption and Quality of Recovery‐15 will be assessed with histograms and Q–Q plots and analyzed using *t*‐tests or Mann–Whitney *U* tests. Pain intensity will be analyzed with repeated measures ANOVA or linear mixed‐effects models. Differences in frequencies will be analyzed using chi‐squared or Fisher's exact tests, as appropriate. An alpha level of 0.05 is used to determine statistical significance. We predefine the minimal clinically relevant difference between the groups in the total opioid consumption as 15 mg of oral MME [15]. Analyses will follow the intention‐to‐treat principle. Missing data will not be imputed or included in the analyses, but the frequency and reasons will be reported in the article. Any changes to the statistical plan will be reported, with the final detailed plan included in the publication.

## 3 Discussion

RCTs on PPB for TKA show heterogeneity in analgesic regimens, anesthesia type, complementary nerve blocks, and local anesthetic volumes (10, 15, and 20 mL), complicating efficacy comparisons [5, 6, 7, 8]. Consensus on the optimal volume is important before comparing PPB to other nerve block techniques targeting the genicular nerves in the popliteal fossa, such as Infiltration between the Popliteal Artery and the Capsule of the Knee (IPACK) and local infiltration analgesia [16, 17]. This trial will be the first clinical investigation on optimal analgesic volume for PPB after TKA.

We have chosen an upper limit of 20 mL of bupivacaine for PPB to avoid exceeding the toxicity margin, as additional local anesthetics are required for the femoral triangle block and spinal anesthesia. We refrained from including a PPB placebo group, as a previous blinded RCT in our hospital settings has already compared the femoral triangle block with and without the addition of PPB [5]. Additionally, only patients receiving spinal anesthesia will be included to avoid potential confounding of postoperative opioid consumption by general anesthesia [18].

Our outcomes are selected to engage traditional domains in the research field of acute postoperative pain, including postoperative opioid consumption, pain intensity at rest and during activity, and physical function. However, we have also included a patient‐reported outcomes measurement to address the quality of recovery from the patient's perspective [19]. The Quality of Recovery‐15 measures five domains including pain, physical comfort, physical independence, psychological support, and emotional state and is translated and validated in a Danish version [14]. The minimal clinically important difference for the Quality of Recovery‐15 is 6 points [19]. There is currently no consensus on the optimal PROM for assessing acute postoperative recovery following TKA; however, the developing process is ongoing [20, 21].

At our hospital's elective surgery center, hospitalization is recommended only for patients without a caregiver at home or those with significant comorbidities. Additionally, patients undergoing surgery in the afternoon may not meet discharge criteria until late in the evening and are often offered overnight hospitalization for comfort, with discharge the following day. As a result, approximately one‐third of our TKA patients are discharged on the day of surgery. To ensure comprehensive inclusion, we enroll both hospitalized patients and outpatients. We do not plan any subgroup analysis between the two groups, as we believe they will be similar.

A potential limitation of this trial is the accuracy of opioid consumption recording, which could be improved using intravenous opioids via a patient‐controlled analgesia pump. However, this would require additional resources and hospitalization of all participants. Instead, we opted for a study design that aligns with our everyday clinical practice.

We hope these results will provide a clinical recommendation for the optimal analgesic volume for PPB, which can optimize postoperative pain management after TKA.

## Author Contributions

Devised protocol: all authors. Made draft for this paper: JKS. Revised and approved draft for this paper: all authors. Approved final manuscript: all authors. The principal investigator and guarantor is JKS. Sponsor is CR.

## Conflicts of Interest

The authors declare no conflicts of interest.

## Data Availability

The data obtained in this study are available from the corresponding author upon reasonable request.

## References

[1] H. J. Gerbershagen , S. Aduckathil , A. J. M. van Wijck , L. M. Peelen , C. J. Kalkman , and W. Meissner , “Pain Intensity on the First Day After Surgery,” Anesthesiology 118, no. 4 (2013): 934–944. 23392233 10.1097/ALN.0b013e31828866b3

[2] S. L. Kopp , J. Børglum , A. Buvanendran , et al., “Anesthesia and Analgesia Practice Pathway Options for Total Knee Arthroplasty: An Evidence‐Based Review by the American and European Societies of Regional Anesthesia and Pain Medicine,” Regional Anesthesia and Pain Medicine 42 (2017): 683–697. 29053504 10.1097/AAP.0000000000000673

[3] P. M. Lavand'homme , H. Kehlet , N. Rawal , and G. P. Joshi , “Pain Management After Total Knee Arthroplasty: PROcedure SPEcific Postoperative Pain ManagemenT Recommendations,” European Journal of Anaesthesiology 39 (2022): 743–757. 35852550 10.1097/EJA.0000000000001691 PMC9891300

[4] S. G. Memtsoudis , C. Cozowicz , J. Bekeris , et al., “Peripheral Nerve Block Anesthesia/Analgesia for Patients Undergoing Primary Hip and Knee Arthroplasty: Recommendations From the International Consensus on Anesthesia‐Related Outcomes After Surgery (ICAROS) Group Based on a Systematic Review and Meta‐Analysis of Current Literature,” Regional Anesthesia and Pain Medicine 46, no. 11 (2021): 971–985. 34433647 10.1136/rapm-2021-102750

[5] J. K. Sørensen , U. Grevstad , P. Jaeger , L. Nikolajsen , and C. Runge , “Effects of Popliteal Plexus Block After Total Knee Arthroplasty: A Randomized Clinical Trial,” Regional Anesthesia and Pain Medicine (2024): 1–7, 10.1136/rapm-2024-105747. 39019501

[6] N. Sakai , T. Adachi , T. Sudani , C. Taruishi , Y. Uematsu , and M. Takada , “Popliteal Plexus Block Compared With Tibial Nerve Block on Rehabilitation Goals Following Total Knee Arthroplasty: A Randomized Non‐Inferiority Trial,” Scientific Reports 14, no. 1 (2024): 23853. 39394446 10.1038/s41598-024-74951-y PMC11470071

[7] K. Stebler , N. Elia , I. Zaccaria , and R. M. Fournier , “Popliteal Plexus Block in Total Knee Arthroplasty: A Single‐Center Randomized Controlled Double‐Blinded Trial,” Regional Anesthesia and Pain Medicine (2024): 1–8, 10.1136/rapm-2024-105782. 39709189 PMC13018729

[8] B. W. Jiang , Y. Guo , M. Y. Yang , et al., “The Analgesic Effect of Continuous Adductor Canal Block Combined With Popliteal Plexus Block for Total Knee Arthroplasty: A Randomized Controlled Trial,” Scientific Reports 14, no. 1 (2024): 27757. 39533094 10.1038/s41598-024-79487-9 PMC11557969

[9] J. K. Sørensen , U. Grevstad , E. Qerama , L. S. Bruun , L. Nikolajsen , and C. Runge , “Effect of Local Anesthetic Volume for Popliteal Plexus Block on Motor Nerve Conduction and Muscle Function in the Leg: A Randomized Clinical Trial in Healthy Volunteers,” Regional Anesthesia and Pain Medicine (2025): 106557, 10.1136/rapm-2025-106557. 40185510

[10] K. F. Schulz , D. G. Altman , and D. Moher , “CONSORT 2010 Statement: Updated Guidelines for Reporting Parallel Group Randomised Trials,” BMJ (Online) 340, no. 7748 (2010): 698–702. 10.1136/bmj.c332 PMC2844940 20332509

[11] D. R. Kerr and L. Kohan , “Local Infiltration Analgesia: A Technique for the Control of Acute Postoperative Pain Following Knee and Hip Surgery—A Case Study of 325 Patients,” Acta Orthopaedica 79, no. 2 (2008): 174–183. 18484242 10.1080/17453670710014950

[12] S. Bjørn , T. D. Nielsen , B. Moriggl , R. Hoermann , and T. F. Bendtsen , “Anesthesia of the Anterior Femoral Cutaneous Nerves for Total Knee Arthroplasty Incision: Randomized Volunteer Trial,” Regional Anesthesia and Pain Medicine 45, no. 2 (2020): 107–116. 10.1136/rapm-2019-100904 31826920

[13] R. W. Bohannon , “Daniels and Worthingham's Muscle Testing: Techniques of Manual Examination,” Physical Therapy 83, no. 2 (2003): 187.

[14] J. Kleif , H. M. Edwards , R. Sort , J. Vilandt , and I. Gögenur , “Translation and Validation of the Danish Version of the Postoperative Quality of Recovery Score QoR‐15,” Acta Anaesthesiologica Scandinavica 59, no. 7 (2015): 912–920. 25867135 10.1111/aas.12525

[15] A. P. H. Karlsen , J. Laigaard , C. Pedersen , et al., “Minimal Important Difference in Postoperative Morphine Consumption After Hip and Knee Arthroplasty Using Nausea, Vomiting, Sedation and Dizziness as Anchors,” Acta Anaesthesiologica Scandinavica 68, no. 5 (2024): 610–618. 38380438 10.1111/aas.14388

[16] L. Andersen and H. Kehlet , “Analgesic Efficacy of Local Infiltration Analgesia in Hip and Knee Arthroplasty: A Systematic Review,” British Journal of Anaesthesia 113 (2014): 360–374. 24939863 10.1093/bja/aeu155

[17] S. K. Sinha , A. Clement , and A. M. Surette , “Infiltration Between the Popliteal Artery and Capsule of the Knee (iPACK): Essential Anatomy, Technique, and Literature Review,” in Current Anesthesiology Reports (Springer, 2019), 474–478.

[18] A. Koutp , G. Hauer , L. Leitner , et al., “Less Induction Time and Postoperative Pain Using Spinal Anesthesia Versus General Anesthesia With or Without the Use of Peripheral Nerve Blocks in Total Knee Arthroplasty,” Journal of Arthroplasty 39, no. 4 (2024): 904–909. 37852447 10.1016/j.arth.2023.10.018

[19] P. S. Myles and D. B. Myles , “An Updated Minimal Clinically Important Difference for the QoR‐15 Scale,” Anesthesiology 135 (2021): 934–935. 34543410 10.1097/ALN.0000000000003977

[20] S. Bigalke , T. V. Maeßen , K. Schnabel , et al., “Assessing Outcome in Postoperative Pain Trials: Are We Missing the Point? A Systematic Review of Pain‐Related Outcome Domains Reported in Studies Early After Total Knee Arthroplasty,” Pain 162 (2021): 1914–1934. 33492036 10.1097/j.pain.0000000000002209

[21] J. Vollert , D. Segelcke , C. Weinmann , et al., “Responsiveness of Multiple Patient‐Reported Outcome Measures for Acute Postsurgical Pain: Primary Results From the International Multi‐Centre PROMPT NIT‐1 Study,” British Journal of Anaesthesia 132, no. 1 (2024): 96–106. 38016907 10.1016/j.bja.2023.10.020

